# Alphaflexivirus Genomes in Stony Coral Tissue Loss Disease-Affected, Disease-Exposed, and Disease-Unexposed Coral Colonies in the U.S. Virgin Islands

**DOI:** 10.1128/mra.01199-21

**Published:** 2022-02-17

**Authors:** A. J. Veglia, K. Beavers, E. W. Van Buren, S. S. Meiling, E. M. Muller, T. B. Smith, D. M. Holstein, A. Apprill, M. E. Brandt, L. D. Mydlarz, A. M. S. Correa

**Affiliations:** a Department of BioSciences, Rice University, Houston, Texas, USA; b Department of Biology, University of Texas, Arlington, Texas, USA; c Center for Marine and Environmental Studies, University of the Virgin Islands, St. Thomas, Virgin Islands, USA; d Mote Marine Laboratory, Sarasota, Florida, USA; e Department of Oceanography and Coastal Sciences, Louisiana State University, Baton Rouge, Louisiana, USA; f Woods Hole Oceanographic Institution, Woods Hole, Massachusetts, USA; KU Leuven

## Abstract

Stony coral tissue loss disease (SCTLD) is decimating Caribbean corals. Here, through the metatranscriptomic assembly and annotation of two alphaflexivirus-like strains, we provide genomic evidence of filamentous viruses in SCTLD-affected, -exposed, and -unexposed coral colonies. These data will assist in clarifying the roles of viruses in SCTLD.

## ANNOUNCEMENT

**FIG 1:**
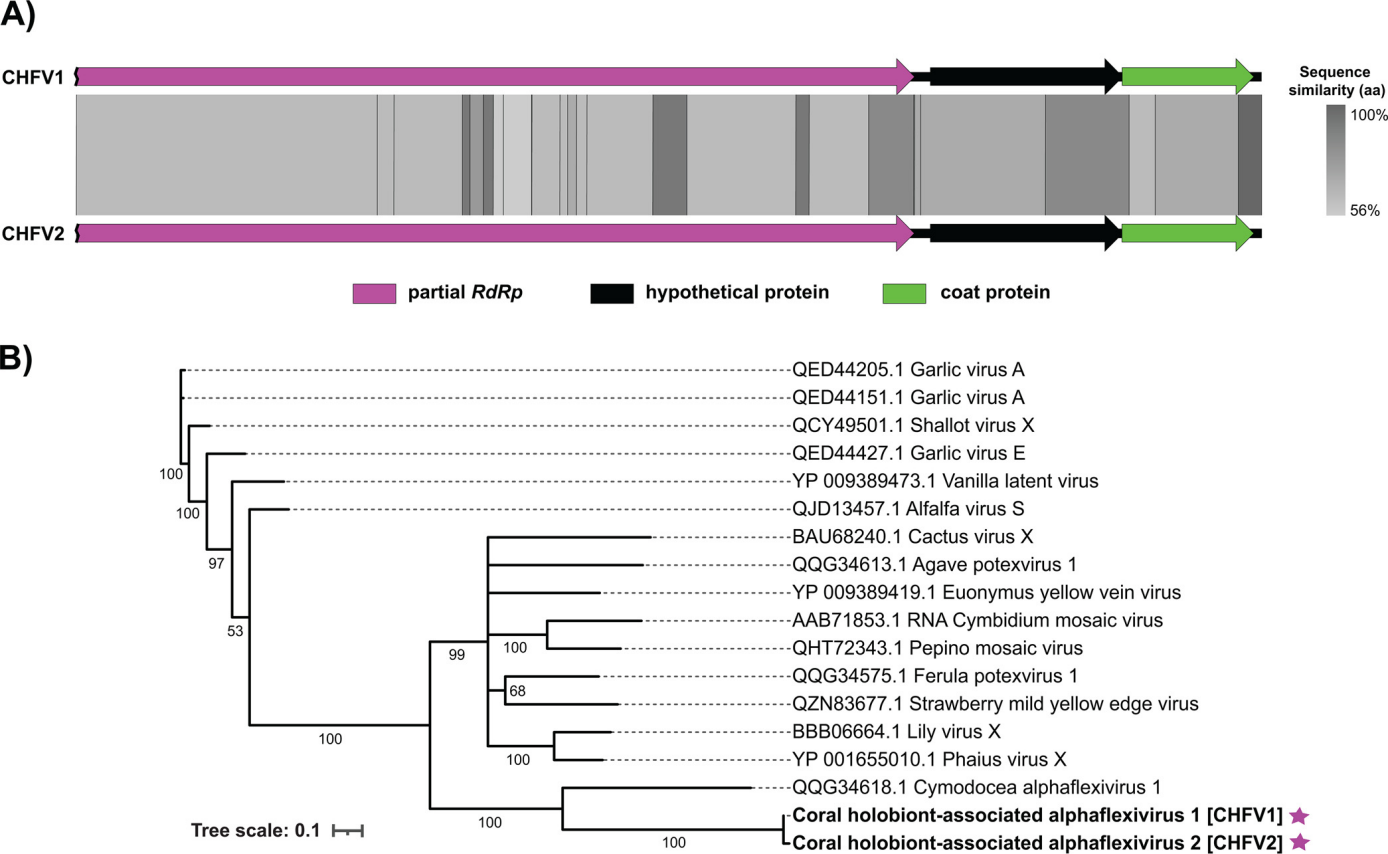
(A) Visualized tBLASTx pairwise alignment of the two coral holobiont-associated alphaflexivirus (CHFV) genomes reported in this study. The arrows represent the predicted genes; the arrow color corresponds to the annotation type. The gray-scale shading between the two genomes represents the percent amino acid (aa) sequence similarity. (B) Maximum likelihood phylogeny generated from translated alphaflexivirus RdRp amino acid sequences from the CHFVs (purple stars) reported in this study, as well as previously described plant-associated alphaflexiviruses. Translated alphaflexivirus RdRp amino acid sequences were aligned using MUSCLE v5 (36) and trimmed using trimAl (37). The phylogeny was constructed using IQTREE v2 (38) with the LG+I+G4 substitution model (determined by ModelFinder [39]), and support was assessed using 1,000 nonparametric bootstrap replicates. The tree was visualized using the Interactive Tree of Life v5 (40); branches with bootstrap support values of <50 were collapsed. The tree scale indicates the number of amino acid substitutions per site.

Viral infections of endosymbiotic dinoflagellates (family Symbiodiniaceae) within coral tissues are hypothesized to play a role in stony coral tissue loss disease (SCTLD) (1), a widespread disease that affects Caribbean stony corals (2–4). Here, we present high-quality draft genome sequences for two viruses in the family *Alphaflexiviridae*, coral holobiont-associated alphaflexvirus (CHFV) 1 and 2 (Fig. 1A), that were assembled from metatranscriptomes from SCTLD-affected, SCTLD-exposed, and control (unexposed) coral holobionts sampled during a SCTLD transmission experiment (5). The field collections were authorized by the Department of Planning and Natural Resources Coastal Zone Management under permit number DFW19057U.

Tissue samples were harvested from 12 frozen fragments of three coral species (Montastraea cavernosa, Porites astreoides, and Pseudodiploria strigosa) collected from St. Thomas, U.S. Virgin Islands (Table 1). Total RNA was extracted using the RNAqueous-4PCR total RNA isolation kit (Invitrogen, Life Technologies AM1914). Tissues were lysed using a refrigerated Qiagen TissueLyser II microcentrifuge at 30 oscillations per second for 30 s. The elution stage consisted of two consecutive 30-μL elutions. Contaminating DNA and chromatin were removed from the total RNA using the Ambion DNase I (RNase-free) kit (Invitrogen, Life Technologies AM2222). Samples were preprocessed by Novogene Co., Ltd. (Davis, CA, USA) for mRNA enrichment using polyA tail capture; the mRNA libraries underwent 150-bp, paired-end sequencing on an Illumina NovaSeq 6000 instrument using the NEBNext Ultra II RNA library prep kit.

**TABLE 1:** Sample information and RNA sequencing results for libraries with reads that contributed to the generation of coral holobiont-associated alphaflexivirus genome assemblies (CHFV1 and CHFV2)

Coral species^*a*^	Sample ID	SRA accession no.	No. of raw reads (millions)	No. of cleaned reads (millions)	No. of noncoral/non-Symbiodiniaceae reads (millions)^*b*^	Colony health status^*c*^	No. of reads mapped to CHFV1	No. of reads mapped to CHFV2
*Montastraea cavernosa*	Mcav_c3	SRR17230326	72.450238	72.148886	19.297152	Control	2,872	142
	Mcav_c6	SRR17230325	43.625450	43.554690	12.021936	Control	780	46
	Mcav_c7	SRR17230323	61.253870	60.945362	17.604312	Control	300	3,524
	Mcav_d2	SRR17230322	58.179614	57.916530	15.643962	Disease exposed	26	152
	Mcav_d3	SRR17230321	55.231168	54.982506	15.468590	Disease affected	834	92
	Mcav_d4	SRR17230320	55.800824	55.586294	15.306024	Disease exposed	16	12
	Mcav_d6	SRR17230319	51.396670	50.957730	15.184224	Disease affected	3,282	142
	Mcav_d8	SRR17230318	62.688392	62.423220	18.170028	Disease exposed	34	56
*Porites astreoides*	Past_c6	SRR17230317	56.484214	56.032382	12.568462	Control	16	16
	Past_d4	SRR17230316	62.052534	61.792644	13.390588	Disease exposed	316	72
	Past_d6	SRR17239955	38.818898	38.603064	8.295652	Disease affected	16	0
*Pseudodiploria strigosa*	Pstrig_d5	SRR17230324	55.559408	55.277124	20.791874	Disease exposed	4,736	1,422

a On shallow reefs in the U.S. Virgin Islands, *M. cavernosa* typically harbors Symbiodiniaceae in the genus *Cladocopium*, *P. astreoides* typically harbors Symbiodiniaceae in the genus *Symbiodinium*, and *P. strigosa* is typically dominated by Symbiodiniaceae in the genus *Breviolum* (6, 7) but can also be dominated by *Cladocopium* symbionts (8).

b Reads not mapping to coral or Symbiodiniaceae transcriptomes and retained for further analysis using BBSplit (within BBMap v38.90) (9). A genome-guided *M. cavernosa* transcriptome was generated using the draft genome from reference 10, and *de novo Porites astreoides* and *Pseudodiploria strigosa* transcriptomes were assembled using Trinity v2.11.0 (11). These reference transcriptomes were generated by the Mydlarz lab (University of Texas at Arlington, Arlington, TX, USA) for internal use but will be made available upon request. Symbiodiniaceae transcriptomes representing the genera *Symbiodinium*, *Breviolum*, *Cladocopium*, and *Durusdinium* were sourced from reference 12, “Kb8 Sequences” (http://medinalab.org/zoox/), reference 13, “*S. minutum*” (http://zoox.reefgenomics.org/download/), reference 14, “Clade C1 Symbiodinium” (http://ssid.reefgenomics.org/download/), and reference 15, “Dtrenchii_rnaseq_assembly_v1.0” (https://datadryad.org/stash/dataset/doi:10.5061/dryad.12j173m), respectively.

c “Disease-affected” colony health status indicates corals that showed active lesions at the time of sampling; “disease-exposed” indicates coral fragments that were exposed to SCTLD but showed no signs of disease by the end of the experiment; “control” indicates that fragments were never exposed to SCTLD and never developed lesions during the course of the experiment.

All bioinformatic tools were run using default parameters unless otherwise specified. BBSplit (BBMap v38.90) was used to map quality-filtered (fastp v0.20.1 [16]) reads to coral or Symbiodiniaceae transcriptomes (9) and generate three read files: (i) coral, (ii) Symbiodiniaceae, and (iii) noncoral/non-Symbiodiniaceae. Noncoral/non-Symbiodiniaceae reads were combined and normalized using BBnorm.sh within BBMap (Table 1). Normalized reads were assembled using the program TransPi (17). Multiple assemblies were generated using rnaSPADES v3.14.0 (kmer: 75,85,91,107 nucleotides) (18), Trans-ABySS v2.0.1 (kmer: 25,35,55,75,85 nucleotides) (19), SOAPdenovo-Trans v1.03 (kmer: 25,35,55,75,85 nucleotides) (20), Trinity v2.9.1 (kmer: 35 nucleotides) (11), and Velvet v1.2.12/Oases v0.2.09 (kmer: 65,71,81,87,91,97,101 nucleotides) (21, 22). The multiple assemblies were concatenated into a single file, and the EvidentialGene *tr2aacds* pipeline v2019.05.14 (23, 24) was used to collapse duplicates and remove misassembled contigs from the assembly file. VirSorter2 (25) was used to detect RNA viruses from the nonredundant metatranscriptome-assembly file (minimum length, 300 nucleotides). Viral genomes similar to known members of the *Alphaflexiviridae* were identified by aligning translated open reading frames (ORFs) to the proteic version of the Reference Virus Database (26, 27) with DIAMOND BLASTx v2.0.11.149 in “ultra-sensitive” mode (28, 29). Cenote-Taker 2 (30) was used to annotate identified viral genomes with similarity to the *Alphaflexiviridae* and calculate the genome coverage using the normalized reads. The alphaflexivirus read count per sample library was estimated by mapping nonnormalized reads to the nonredundant assembly using bowtie2 (31) with the align_and_estimate_abundance.pl script (11; Table 1).

The CHFV1 and CHFV2 genomes are linear, share 85.9% genome-wide nucleotide identity, and are 6,228 and 6,227 nucleotides long with 42.4% and 42.0% G+C content, respectively. Coverages for the CHFV1 and CHFV2 assemblies are estimated at 334.9× and 123.4×, respectively. CheckV (32) was used to identify the genomes as high quality with 90% completeness (average amino acid identity-based [medium-confidence]). Visualization of a tBLASTx (33) pairwise alignment between the CHFV genomes was conducted using Easyfig (34) and depicted the genomes’ three shared ORFs (Fig. 1A). The closest relative of the CHFV genomes, as determined using Cenote-Taker 2, is strawberry mild yellow edge virus (NCBI protein accession number NP_620642.1) (35), sharing ∼33.5% amino acid similarity for the RNA-dependent RNA polymerase (RdRp) (ORF1).

A phylogenetic tree was generated from translated RdRp sequences from the two CHFVs and 16 plant-associated alphaflexiviruses (Fig. 1B). The CHFV replicase sequences formed a clade with the RdRp sequence of an unclassified alphaflexivirus that infects Cymodocea nodosa seagrass (Fig. 1B).

The CHFV genomes reported here constitute genomic-based evidence of filamentous viruses from coral colonies. Quantitative PCR primer sets can be developed from these genome assemblies to support the critical next step of characterizing the presence/absence and abundance of coral holobiont-associated alphaflexiviruses across coral colonies, to further clarify the potential role of viruses in SCTLD.

### Data availability.

Coral holobiont-associated alphaflexivirus 1 and 2 have been deposited at NCBI’s GenBank (accession numbers OM030231 and OM030232). The raw reads from the transcriptome sequencing (RNA-Seq) libraries were deposited at NCBI’s Sequence Read Archive (SRA) under BioProject accession number PRJNA788911 (Table 1).
